# The role of AMPK in metabolism and its influence on DNA damage repair

**DOI:** 10.1007/s11033-020-05900-x

**Published:** 2020-10-18

**Authors:** Michał Szewczuk, Karolina Boguszewska, Julia Kaźmierczak-Barańska, Bolesław T. Karwowski

**Affiliations:** DNA Damage Laboratory of Food Science Department, Faculty of Pharmacy, Medical University of Lodz, Ul. Muszynskiego 1, 90-151 Lodz, Poland

**Keywords:** AMPK, DNA damage, DNA repair, 8-oxoguanine glycosylase

## Abstract

One of the most complex health disproportions in the human body is the metabolic syndrome (MetS). It can result in serious health consequences such as type 2 diabetes mellitus, atherosclerosis or insulin resistance. The center of energy regulation in human is AMP-activated protein kinase (AMPK), which modulates cells’ metabolic pathways and protects them against negative effects of metabolic stress, e.g. reactive oxygen species. Moreover, recent studies show the relationship between the AMPK activity and the regulation of DNA damage repair such as base excision repair (BER) system, which is presented in relation to the influence of MetS on human genome. Hence, AMPK is studied not only in the field of counteracting MetS but also prevention of genetic alterations and cancer development. Through understanding AMPK pathways and its role in cells with damaged DNA it might be possible to improve cell’s repair processes and develop new therapies. This review presents AMPK role in eukaryotic cells and focuses on the relationship between AMPK activity and the regulation of BER system through its main component—8-oxoguanine glycosylase (OGG1).

## Introduction

The MetS (metabolic syndrome) is a combination of specific systemic symptoms associated with improperly functioning metabolic pathways, failures of energy utilization and energy storage mechanisms. Hypertension, hyperglycemia, central obesity and low level of serum high-density lipoprotein are main characteristics [1]. It is assumed that MetS-related diseases, as well as MetS itself, result from unhealthy dietary habits and in some extent from genetic predispositions. The more advanced the state of disease development is, the more complex the treatment approach and lifestyle changes have to be. MetS and accompanying diseases increase patient’s risk of further health problems, including many types of cancer [2]. MetS is also associated with oxidative stress, ROS (reactive oxygen species) generation and inflammation in many organs, therefore it is one of factors causing DNA damage [1–3].

T2DM (type 2 diabetes mellitus) has become one of the most common human health problems with around 422 million cases among adults worldwide. It is estimated, that the global prevalence will increase by 55% up to the year 2035 [4]. A crucial role in T2DM is played by IR (insulin resistance). Reduced sensitivity to insulin and impaired glucose uptake leads to an increased level of total blood glucose. Since IR primarily affects skeletal muscle, liver and adipose tissue, it has a significant impact on energy-consuming metabolic pathways [1, 3, 5]. Many of existing biochemical pathways involved in human energy generation and consumption are proved to be modulated by AMPK (AMP-activated protein kinase)—key molecular target in treatment of metabolic syndrome and related abnormalities [5–7]. Negative effects of disturbed cellular energy balance on the cell functionalities are widely described. Prolonged hyperglycemia is responsible for ROS overgeneration and may be followed by contractile dysfunctions in diabetic heart. Therefore, main enzymes taking part in ROS generation, such as NADPH oxidase are considered targets in treating diabetes-induces cardiovascular issues [8, 9]. Elevated ROS production is also observed in cells expressing oncogenic Ras protein. Ras is expressed in tumors to modulate cell growth and differentiation. Metformin is known to decrease ROS production in Ras-expressing human fibroblasts through AMPK-dependent mechanism [10]. Moreover, metformin and berberine exhibit anti-inflammatory properties in intestinal epithelium through indirect AMPK activation. It is associated with ROS overgeneration in mitochondria. ROS overgeneration is considered the main source of metabolic dysfunctions of various types of tissues and has a pivotal role in induction of damage within the structure of nucleic acids. To understand the effect of ROS production on metabolic pathways and DNA structure, it is needed to understand molecular mechanisms ongoing inside mitochondria and the AMPK response pathways in details.

The authors believe it is worth exploring the connections of the AMPK and its metabolic roles with protecting the genome integrity in the cells prone to ROS action. The first part of this paper presents characteristics of AMPK and the role it plays in metabolic pathways. Further, the mitochondrial ROS generation is described followed by OGG1 (8-oxoguanine glycosylase) as the enzyme recognizing the most abundant oxidative damage and BER (base excision repair) system as the pathway repairing mitochondrial damage. The last part explains how AMPK influences DNA damage repair in the context of metabolism maintenance.

## Structure and activation of AMPK

### AMPK structure

AMPK (5′ adenosine monophosphate-activated protein kinase) is an eukaryotic enzyme, a representative of transferase class, kinase subclass—EC. 2.7.11.31. Its main function is regulation of energy homeostasis through controlling uptake of glucose and fatty acids by the cell. AMPK is a heterotrimeric complex containing three subunits—alpha (α) which plays a catalytic role, beta (β) and gamma (γ) which are regulatory subunits. Until now, isoforms of each subunit have been identified in mammals—α1, α2, β1, β2, and γ1–γ3. Therefore, AMPK may occur in 12 different isoforms depending on the type of the cell [11, 12].

Catalytic subunit α contains C-terminus, which is essential to create a stable complex with other subunits. Its isoforms (α1, α2) have similar substrate specificity but in some cases α1 is more likely to be present on the cell membrane surface, while α2 is found inside the nucleus [13]. Subunit α also contains the Ser/Thr kinase domain on its N-terminus which is the main component of the enzyme. It contains T-loop fragment with Thr172—the main target of the enzyme activation mechanism (phosphorylation/dephosphorylation of Thr172). Before enzyme activation, subunit α remains inactive [12–14]. β subunit is the core of AMPK. It binds the other subunits with its C-terminus, while its N-terminus is responsible for carbohydrate binding (e.g. glycogen). It takes part in maintaining level of cellular glycogen. Therefore, AMPK may lead to inhibition of glycogen synthesis in muscles and the liver [12, 14]. Subunit γ takes part in enzyme activation. It is connected to β subunit by its N-terminus. This subunit is a place of AMPK main ligand binding—adenosine phosphates. Universal binding occurs competitively through four CBS (cystathione β-synthase) motifs, which are located close to the N-terminus [15]. CBS motifs work in pairs as Bateman Domains (BD) (BD1: CBS1/CBS2, BD2: CBS3/CBS4). Three binding sites are known: CBS1, CBS3 and CBS4. CBS2 does not bind substrates due to Asp to Arg replacement. Although adenosine phosphates are considered the main ligands of AMPK, other adenine derivatives can also be bound by γ subunit. For example, NADH and NADPH molecules may be bound by CBS3 motif, yet there is no clear evidence it has any physiological relevance in terms of AMPK activation [16, 17].

### AMPK activation

Binding a substrate causes an allosteric change in AMPK structure and AMPK activity increases. It occurs through one of three mechanisms: (1) direct allosteric activation with AMP as the only substrate (up to 10-fold activity increase), (2) phosphorylation of Thr172, (3) prevention of Thr172 dephosphorylation; (2) and (3) can occur in the presence of AMP and ADP. All three mechanisms take place alternatively but in non-stress conditions only phosphorylation of Thr172 leads to fully active state of the enzyme with over 1000-fold activity increase [12, 18, 19]. Phosphorylation and dephosphorylation of Thr172 depend on the changes in AMPK structure—access to the T-loop is controlled allosterically by AMP binding. Thr172 can be phosphorylated by kinases and dephosphorylated by phosphatases. LKB1 (liver kinase B1, also known as Ser/Thr kinase 11 (STK11)) demonstrated the highest affinity for the allosteric structure of AMPK. When *STK11* is mutated it may lead to Peutz-Jeghers Syndrome and many types of cancer [20, 21]. Two different AMPK activation mechanisms may occur simultaneously. Allosteric activation of AMPK increases its affinity to kinases [22] and at the same time may decrease its affinity to phosphatases (e.g. protein phosphatase 2C (PP2C)), protecting the enzyme from dephosphorylation [23]. It is hypothesized that with low concentrations of AMP in the cell, phosphorylation and dephosphorylation of Thr172 take place alternately at all times. Only the elevation of AMP level results in the inhibition of dephosphorylation, thanks to which the enzyme remains active and can work properly [24].

Moreover, AMPK could be indirectly activated by high concentration of intracellular Ca^2+^, according to studies using *STK11* knockout HeLa cancer cells [21]. Calcium cations activate a specific class of kinases—CaMKK kinases (Calcium/Calmodulin-dependent kinase kinase). One member of this class in particular—CaMKK2—is able to phosphorylate Thr172 and may be activated by increase of Ca^2+^ and ROS concentrations inside the cell [25, 26]. It is found that presence of the piperine—an alkaloid widely present in food, can contribute to an increase of Ca^2+^ and ROS concentrations inside the cell, hence activation of CaMKKβ [5].

### Types of AMPK activators

Since many AMPK activators have been considered as therapeutic agents, they were widely studied for their properties and possible applications. AMPK activators are divided into three groups basing on their mechanism of action.

**Fig. 1:**
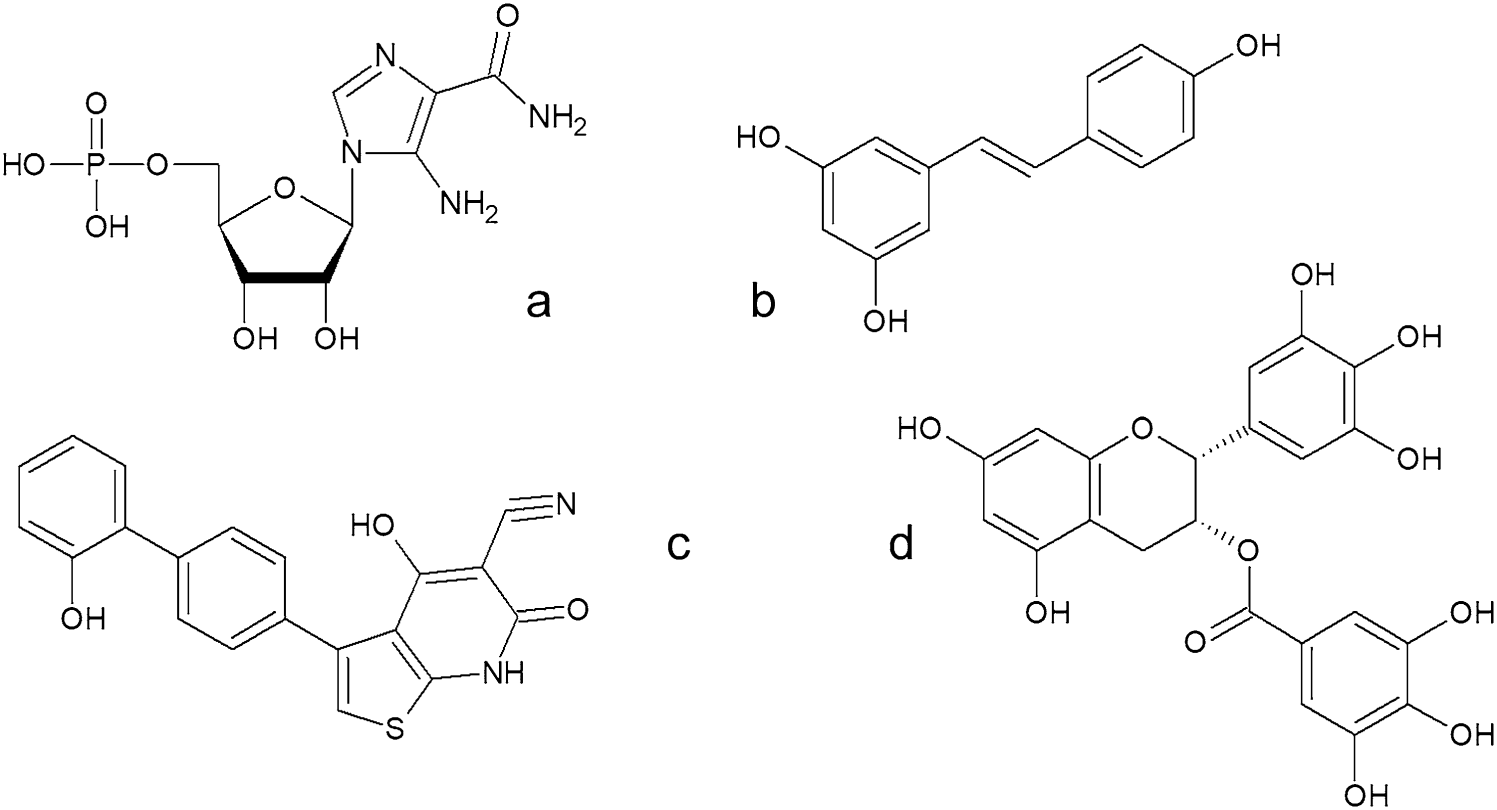
Structure of the exemplary AMPK activators: **a** AICAR (acadesine, 5-aminoimidazole-4-carboxamide ribonucleoside), **b** resveratrol, **c** thienopyridone A769662, **d** EGCG (epigallocatechin gallate)

First group of AMPK activators consists of AMP analogs (competitive activators) and are present in the cell mostly in the form of prodrugs. The first known pharmacological compound capable of mimicking an impact of AMP on AMPK in vivo was AICAR (acadesine, 5-aminoimidazole-4-carboxamide ribonucleoside). AICAR is known as a target for treatment of acute lymphoblastic leukemia [27]. Studies performed on rat hepatocytes proved that AREBP protein (AICAR responsive element binding protein) is phosphorylated by AMPK on Ser470—it blocks glucose production and its uptake from blood becomes inefficient [28]. On the other hand, this process became questionable after publication of results showing that AICAR directly inhibits fructose-1,6-bisphosphatase in hepatocytes lacking AMPK [29]. Despite contradictory results, the latest research seem to support the mutual impact of AICAR and AMPK on hepatic glucose production [30]. The second group of AMPK activators are compounds binding directly to β subunit of AMPK. The first known compound in this group was thienopyridone A769662. It also mimics actions of AMP [11, 31]. The third group of AMPK activators consists of compounds that activate the enzyme indirectly. They may affect the AMP/ATP ratio, disturb respiratory chain reactions, carbohydrate uptake or ATP generation. These are mostly polyphenols and alkaloids such as resveratrol, metformin, curcumin, piperine, EGCG (epigallocatechin gallate) and caffeic acid (Fig. 1) [5, 32–36].

## AMPK and its influence on metabolism

Metabolism is referred to as every chemical reaction responsible for its all biological operations carried out by properly functioning cell. Metabolic pathways are anabolic or catabolic. Catabolism reactions breakdown large molecules to obtain energy. Released ATP is consumed by anabolic reactions and supplied to organelles. The conversion of simple chemical compounds occurs into complex ones that are essential for cell growth [37, 38]. To keep the cell operating total energy balance inside the cell organelles (balance between catabolic and anabolic reactions) needs to remain unchanged. AMPK plays a crucial role in the maintenance of energy balance. It acts as a sensor of the internal adenosine phosphates level. Once activated, AMPK inhibits energy-consuming pathways (e.g. fatty acid synthesis) and affects energy-generating pathways (e.g. glycolysis and glycogenolysis) [4]. Influence of AMPK on human metabolism has been characterized in detail over the years [7, 18, 39]. Special attention was given to muscles due to their continuous energy demand and intensified regeneration process after physical exercise. Additionally, impact of AMPK on other cell types (adipocytes, liver, pancreas, kidney) has been studied [40]. AMPK plays a role in all main metabolic pathways concerning glucose uptake, carbohydrates and lipid metabolism.

### Glucose uptake

Maintaining proper carbohydrates level inside the cell comes down to modulation of glucose level. AMPK modulates glucose uptake from capillaries. Phosphorylation of IRS-1 (insulin receptor substrate 1) protein at Ser794 occurs and affects PKB (protein kinase B, also known as Akt) signaling. PKB phosphorylates its substrate—AS160 protein (PKB substrate of 160 kDa). It detaches from the surface of intracellular vesicles causing the release and translocation of GLUT4 transporter (glucose transporter type 4) to the plasma membrane. GLUT4 transports glucose into the cells, where the glycolysis commences. There is evidence indicating that activated AMPK stimulates also PKB (phosphorylation at Ser789). However, actions of AMPK and PKB are antagonistic and the process is still under investigation [27, 41–43].

## Metabolism of carbohydrates

AMPK affects three fundamental pathways regulating glucose homeostasis: gluconeogenesis, glycolysis and glycogenolysis. In gluconeogenesis, activation of AMPK leads to inhibition of the pathway. AMPK impacts activity of transcription factors: CBP (CREB binding protein), CRTC2 (CREB regulated transcription coactivator 2) and SHP (small heterodimer partner) receptor. They regulate synthesis of G6P (glucose-6-phosphatase) and PEPCK (phosphoenolpyruvate carboxykinase), which are crucial for the course of gluconeogenesis. Current in vitro studies claim that inhibition of gluconeogenesis in human cancer cells by itraconazole is mediated by AMPK pathway [44, 45]. Moreover, suppression of AMPK by FSH (follicle-stimulating hormone) in mice liver enhances transcription of *PEPCK* and *G6P* via CRTC2 [46]. Stimulation of glycolysis by AMPK involves activation of PFK-2 (phosphofructokinase 2, EC 2.7.1.105) by its phosphorylation at Ser466. PFK-2 catalyzes the conversion of fructose-6-phosphate into fructose-1,6-bisphosphate, which is a third stage of glycolysis. It is worth noting that effect of AMPK on glycolysis is cell type-specific—only two of the four isoforms of PFK-2 are activated by AMPK [47, 48]. AMPK is also involved in regulation of catabolic glycogenolysis. During not strenuous muscle exercise active AMPK phosphorylates numerous molecules of glycogen synthase (EC 2.4.1.11) which are attached to the glycogen surface. Activity of glycogen synthase is inhibited; thus, the synthesis of glycogen is no longer possible. Instead, glucose is used as energy supply for muscles [49–51].

### Metabolism of lipids

In energy deficient states, AMPK inhibits catabolic lipid synthesis by reducing the activity of ACC (acetyl-CoA carboxylase, EC 6.4.1.2) [52]. AMPK phosphorylates Ser79, Ser1200 and Ser1215 in ACC isoform 1 (ACC1) and Ser219 in ACC isoform 2 (ACC2). AMPK also stimulates beta-oxidation by activating expression of carnitine acyltransferase I (CPT-1/CAT-1). Acyl-CoA are transformed into acetyl-CoA which is incorporated into the Krebs cycle and oxidized to generate ATP [53]. AMPK inhibits activity of HMG-CoA reductase (3-hydroxy-3-methylguaryl-CoA reductase, EC 1.1.1.88) by its phosphorylation at Ser871. HMG-CoA reductase catalyzes the conversion of HMG-CoA (3-hydroxy-3-methylglutaryl-coenzyme A) into mevalonic acid as one of the steps of the cholesterol synthesis [54]. AMPK in this case blocks cholesterol synthesis. AMPK is also known to indirectly modulate the activity of FAS (fatty acid synthase, EC 2.3.1.85) [55–57]. FAS expression is controlled by SREBP (Sterol regulatory element-binding proteins) and ChREBP (Carbohydrate-responsive element-binding protein) transcription factors, which are modulated by AMPK. The influence of AMPK on human transcription factors, such as SREBP, ChREBP, FOXO family and PPAR (peroxisome proliferator-activated receptor) family has been widely discussed [58–61]. It reveals that the enzyme is able to control cellular metabolism not only by its direct interactions, but also on the genetic level. However, this extensive discussion is not a part of this review.

## AMPK and DNA damage sources in mitochondria

Mitochondria are intracellular double-membraned organelle, responsible for providing energy to living cells. Since the discovery of mitochondria in the early 1840s they are described as fundamental to almost every eukaryotic cell. The energy is generated in the form of ATP, as a result of multiple molecular mechanisms—oxidative phosphorylation and respiratory chain. Although mitochondria are present in cells in large quantities, maintaining the proper functioning of every single mitochondrion is crucial to survival of cell as a whole [62, 63]. Mitochondrial DNA (mtDNA) is an easier target for destructive actions of ROS than nuclear DNA. Lack of arranged structure of histone-assisted chromatin and direct proximity of the electron transport chain are main reasons [64]. Since mitochondria synthesize ATP, the overall energy balance inside the cell gets easily disrupted by mitochondrial dysfunctions due to ROS overgeneration and/or damaged genetic material [25].

### Mitochondrial survival systems

The mitochondria control system is divided into two parts—regulation of mitochondrial fusion/fission dynamics and mitochondrial autophagy (mitophagy). When a mitochondrion is affected by minor stress, it is able to integrate with other healthy mitochondrion in the fusion process. It includes sharing undamaged components and genetic complementation. It allows retrieving lacking genome fragments of the damaged mitochondrion. When a mitochondrion stays under stress conditions for a longer period of time or gets heavily damaged, it is separated from healthy mitochondria in the fission process. Heavily damaged ones are degraded by mitophagy. Their undamaged components are “recycled”—used for fusions or new mitochondria. Fusion/fission requires outer membrane fusion proteins: Mfn1 and Mfn2 (mitofusion protein 1 and 2), the inner membrane fusion protein Opa1 (optic atrophy type 1) and the fission protein Drp1 (dynamin-related protein 1). All of the proteins are activated through phosphorylation by upstream kinases [62, 65, 66]. The fusion/fission system decreases the effect of prolonged stress conditions on mitochondria functionalities. Especially when it comes to the most degenerative type of stress factor—reactive intermediates generated by mitochondria themselves. An imbalance between intracellular generation of reactive intermediates and the cells’ ability to eliminate and detoxify them is called OS (oxidative stress). OS takes place when organism is no longer able to compensate overall damage made by ROS. The most significant source of ROS is oxidative phosphorylation, occurring in mitochondria. Superoxide anion (O_2_^•−^), hydroxyl radical (^•^OH), singlet oxygen (^1^O_2_) and hydrogen peroxide (H_2_O_2_) are generated [67]. In the normal state, cells keep ROS production at lower levels, which is harmless to metabolic pathways. However, in the case of stress occurrence, that level is elevated. ROS generation have an impact on DNA structure and may cause many types of damage. DNA damage includes single- and double-strand breaks (SSB and DSB), base damage or multiple AP-sites (abasic sites) [66, 68, 69]. Apart from ROS activity, strand breaks are also induced by endonucleases (e.g. as part of cell death processes), genotoxic chemicals, anticancer drugs or ionizing radiation [67, 70].

A well-known ROS-induced mutation is oxidation of guanine to 8-oxodG (8-oxo-7,8-dihydro-2′-deoxyguanosine), which demonstrates mutagenic properties. Guanine is the most sensitive nucleobase to oxidative damage due to its low redox potential [69, 71]. Presence of 8-oxodG in DNA may lead to its mispairing with dATP (deoxyadenosine triphosphate) during replication. Therefore, G:::C → T::A transversion occur which cause genomic instability and may initiate or accelerate carcinogenesis. Many studies have shown that cancer tissues exhibit elevated 8-oxodG levels [67, 72].

Among the cellular antioxidant protective agents the NRF-2 (nuclear factor erythroid 2–related factor 2) is considered to be the most important. It is a nuclear transcription factor that binds to ARE (antioxidant-response element) and regulates expression of chemoprotective genes in response to oxidative stress. These genes encode proteins such as HO-1 (heme oxygenase 1) and NQO1 (NADPH:quinine oxidoreductase 1). Inhibition of AMPK and activation of mTORC1(mammalian/mechanistic target of rapamycin complex 1)/raptor is associated with lowered expression of NRF-2 and OGG1 in diabetic mice compared to wild type mice. AMPK activation by AICAR is associated with Ser792 phosphorylation and dissociation of raptor protein (component of mTORC1) from mTORC1 to inhibit its expression. In addition, decrease in mTORC1 expression can lead to significantly higher expression of NRF-2 and OGG1 in AICAR-treated mice compared to non-treated mice. This suggests that AICAR operates through activation of AMPK and subsequent inhibition of mTORC1 through raptor protein. The above can cause increased OGG1 protein expression by upregulation of NFR-2 and decreased oxidative DNA damage [73]. Additionally Duan et al. has shown that butin—an organic flavanone present in plants (*Dalbergia*), facilitates the expression of NRF-2 in rat’s myocardial cells by induction of ARE-mediated expression of antioxidant genes. The proposed mechanism can occur by phosphorylation of AMPK/Akt/GSK-3β pathway. Butin-activated AMPK and Akt induces phosphorylation of GSK-3β (Glycogen synthase kinase 3β) at Ser9 position. It may inhibit GSK-3β–mediated NRF-2 degradation, resulting in its nuclear accumulation and activation of NRF-2 related proteins in myocardial cells [8].

### AMPK—cooperation with mitochondria

**Fig. 2:**
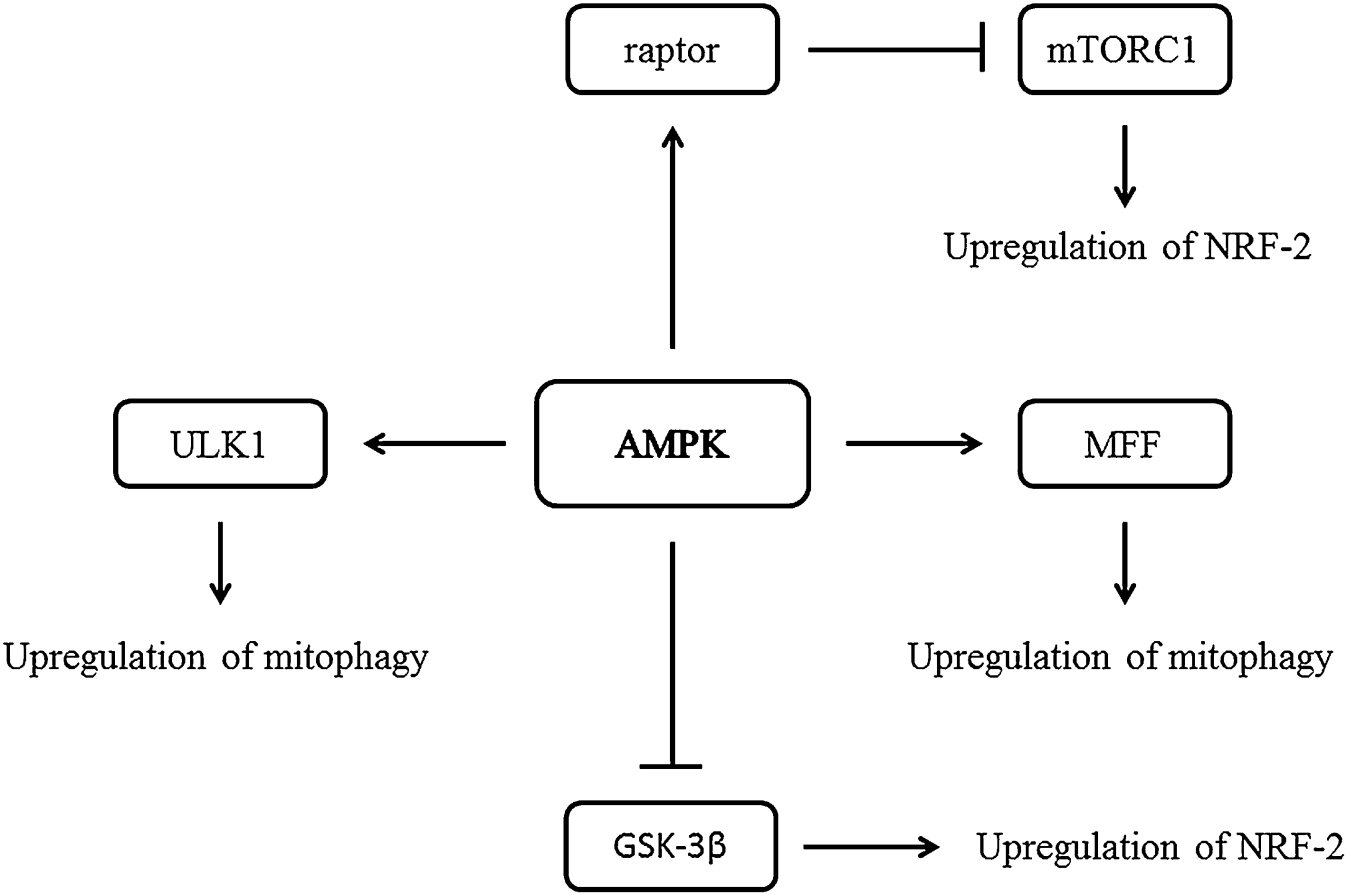
The influence of AMPK on cellular antioxidative mechanisms. Examples of direct and well described actions of AMPK are shown

AMPK plays a key role in maintaining proper level of healthy mitochondria by regulation of fusion/fission and mitophagy mechanisms (Fig. 2). AMPK activation may be dependent on ROS generation. The process called mitochondria uncoupling is based on increased oxygen consumption by oxidative chain reactions. Meanwhile, ATP synthesis by oxidative phosphorylation is inefficient. Uncoupling is mediated by uncoupling proteins (UCPs) located on the surface of the inner mitochondrial membrane. They disturb the electrochemical proton gradient created by mitochondrial oxidative chain. Upon UCPs activation mitochondrial proton pumps start pushing protons out of the matrix to compensate gradient fluctuations. It results in lack of protons inside mitochondria, which leads to intensification of oxidative chain reactions. Mitochondria focus on gradient compensation; thus their ability to ATP generation drastically decreases, resulting in AMPK activation [74–76].

AMPK modulates the activity of transcription factors. One of which, PGC-1α (peroxisome proliferator-activated receptor-γ co-activator 1α), is responsible for mitochondrial biogenesis by enhancing transcription of nuclear-encoded mitochondrial genes. Moreover, AMPK switches on mitophagy by promoting expression of fusion proteins—Mfn1 and Mfn2, the inner membrane fusion protein Opa1 and the fission protein Drp1. A recent study shows that AMPK activation by AICAR enhances mitochondria fragmentation in mouse embryonic fibroblasts and hepatocytes under induced stress conditions. Although the pathway remains unknown, AMPK activity is essential for the mitochondrial control system [62, 63, 66]. Another role of AMPK in mitochondrial dynamics modulation is phosphorylation of MFF protein (mitochondrial fission factor). MFF recruits Drp1 protein from cytosol to mitochondrial surface [66]. Direct phosphorylation of three different variants of MFF at Ser129 has been shown [77]. Moreover, action of AMPK/MFF/Drp1 pathway in human mesenchymal stromal cells (MSC) has been demonstrated [78]. Mitophagy is AMPK-mediated through phosphorylation of ULK1 (unc-51 like autophagy activating kinase 1) protein at Ser555 in human MSC [79], human acute myeloid leukemia stem cells [80] and mouse skeletal muscle [81]. ULK1 takes part in regulation of autophagosomes formation (initial step of mitophagy), hence it may play crucial role in regulation of metabolic actions in response to nutritional stress—e.g. amino acids withdrawal [82].

## AMPK influence on DNA damage repair

### Cell cycle modulation

Besides the regulation of mitochondria biogenesis, one of the most important AMPK-dependent actions, occurring after DNA-damage detection, is induction of autophagy and growth suppression of the cells. In non-stress conditions, the cell cycle and cell growth are modulated by mTOR pathway. The main protein in this process is mTORC1. In order to enhance cell growth, mTORC1 must be activated by RHEB protein (Ras homolog enriched in brain). It is able to activate mTORC1 only if bound to GTPase. Inhibition of cell growth begins with phosphorylation of TSC2 (Tuberous Sclerosis Complex 2, also known as tuberin) by AMPK at Thr1227 and Ser1345. It leads to inhibition of RHEB ability of binding GTPase [83, 84].

In damage response signaling, also ATM (ataxia telangiectasia mutated) Ser/Thr kinase is involved (EC 2.7.1.11). It acts as an upstream kinase for LKB1. ATM is mostly present in the nucleus and becomes activated after DSB detection. However, it may be found in cytoplasm where its activation occurs via OS or ionizing radiation. Activation of ATM occurs through autophosphorylation at Ser1981 and results in activation of DNA repair system or apoptotic pathways. ATM activation leads to LKB1 phosphorylation, which leads to AMPK and TSC2 activation and mTORC1 suppression. MTORC1 is known as natural negative regulator of autophagy, thus as a consequence of mTORC1 suppression, autophagy is induced [85]. ATM is able to phosphorylate AMPK at Thr172 independently of LKB1. In case of serious fluctuations in mitochondrial activity or elevated ROS generation, AMPK may be activated parallelly after its phosphorylation by ATM or LKB1. Moreover, as mentioned above, AMPK is able to directly phosphorylate raptor protein to downregulate its signaling [83, 86, 87]. Since cell growth consumes massive amounts of energy, AMPK is able to modulate energy balance by regulating cell cycle and growth through the mTORC1 pathway, in particular after serious DNA damage is detected.

### Non-homologous end joining (NHEJ)

Another novel function of LKB1 consists of participation in chromatin remodeling during the initial step of DSBs repair by non-homologous end joining (NHEJ). Accumulation of LKB1 at the damage sites is required for the activation of a specific SWI/SNF (switch/sucrose non-fermentable) chromatin remodeling complex. NHEJ is also promoted via LKB1-AMPK-dependent phosphorylation of histone H2B [88]. The initial step of NHEJ process takes place with the association of KU70 and KU80 proteins. They form heterodimeric complex known as KU protein and bind to DNA-PK (DNA-dependent protein kinase) to create active holoenzyme. Active DNA-PK complex mediates the DSBs repair through NHEJ. Additionally DNA-PK can phosphorylate Thr5 and Thr7 residues of chaperone protein HSP90 (heat shock protein 90) subunit alpha, which is responsible for appropriate folding of LKB1 and AMPK proteins. The phosphorylation can lead to HSP90α chaperone function disruption with subsequent negative AMPK folding regulation [89–91]. These findings show the connection of AMPK and proteins involved in DNA repair by NHEJ pathway. Also, KU70 and KU80 proteins are known to interact with particular elements of BER pathway and impair its functioning e.g. they may interact with AP-sites to inhibit APE1 (apurinic/apyrimidinic endonuclease 1) activity. The data on AMPK operation in this particular phenomenon is scarce [92]. AMPK is important for DNA repair machinery; however, further studies are highly demanded in order to clarify its specific roles.

### Base excision repair (BER)

**Fig. 3:**
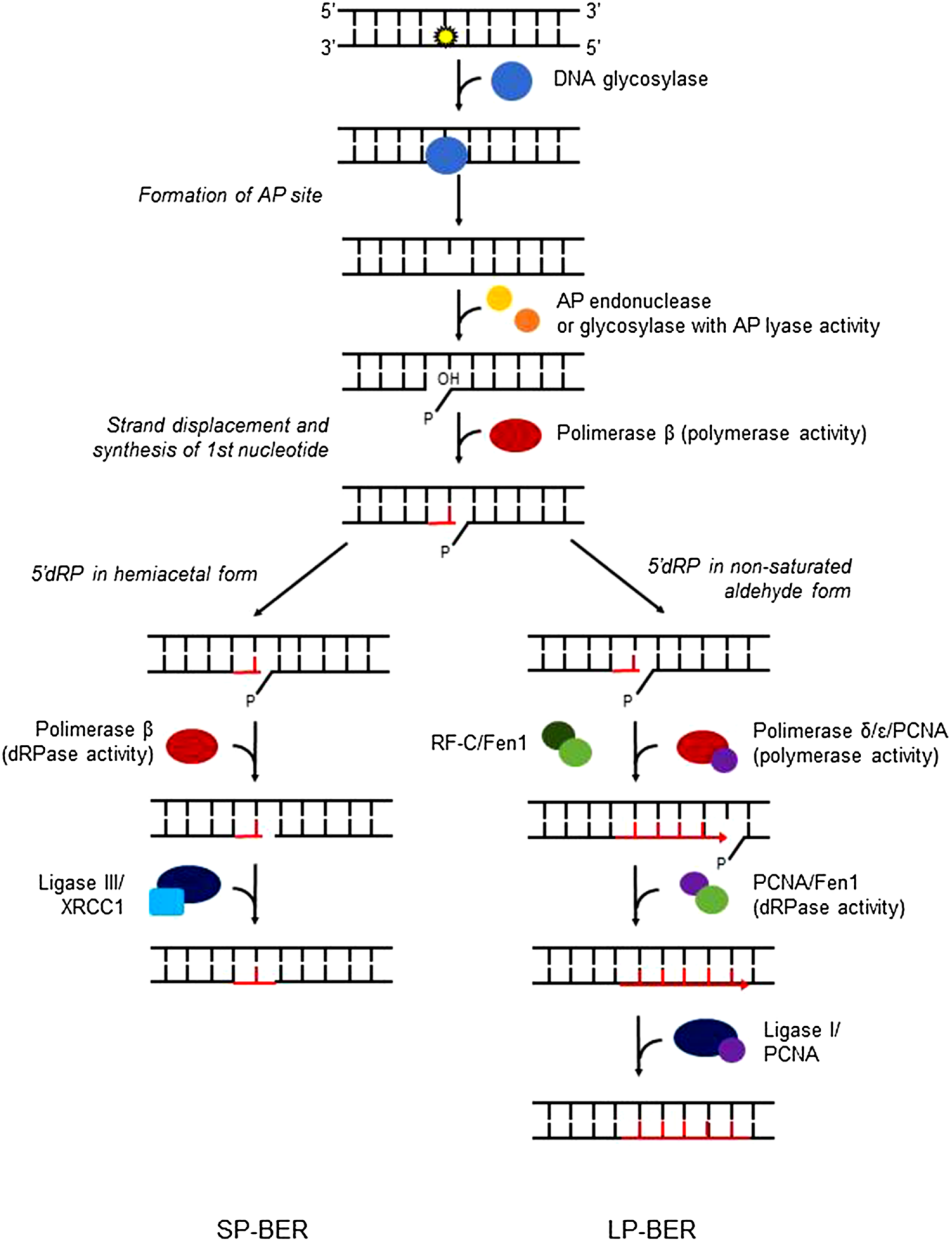
Schematic representation of BER (base excision repair) mechanism. Differences between subsequent stages of long patch BER and short patch BER are presented. The most important proteins are shown

8-oxodG is one of the most abundant DNA damage occurring as a result of oxidative stress [93]. The BER is designed to eliminate the damage through multistep mechanism (Fig. 3). Short patch (SP-BER) and long patch (LP-BER) mechanism are distinguished—they differ in the length of the patch that is repaired: one nucleotide for SP-BER and up to fifteen nucleotides for LP-BER. BER involves a group of specific enzymes: glycosylases recognizing damaged bases, endonucleases excising the damage, polymerases inserting correct nucleotide and ligases nicking the strand [69, 94].

**Fig. 4:**
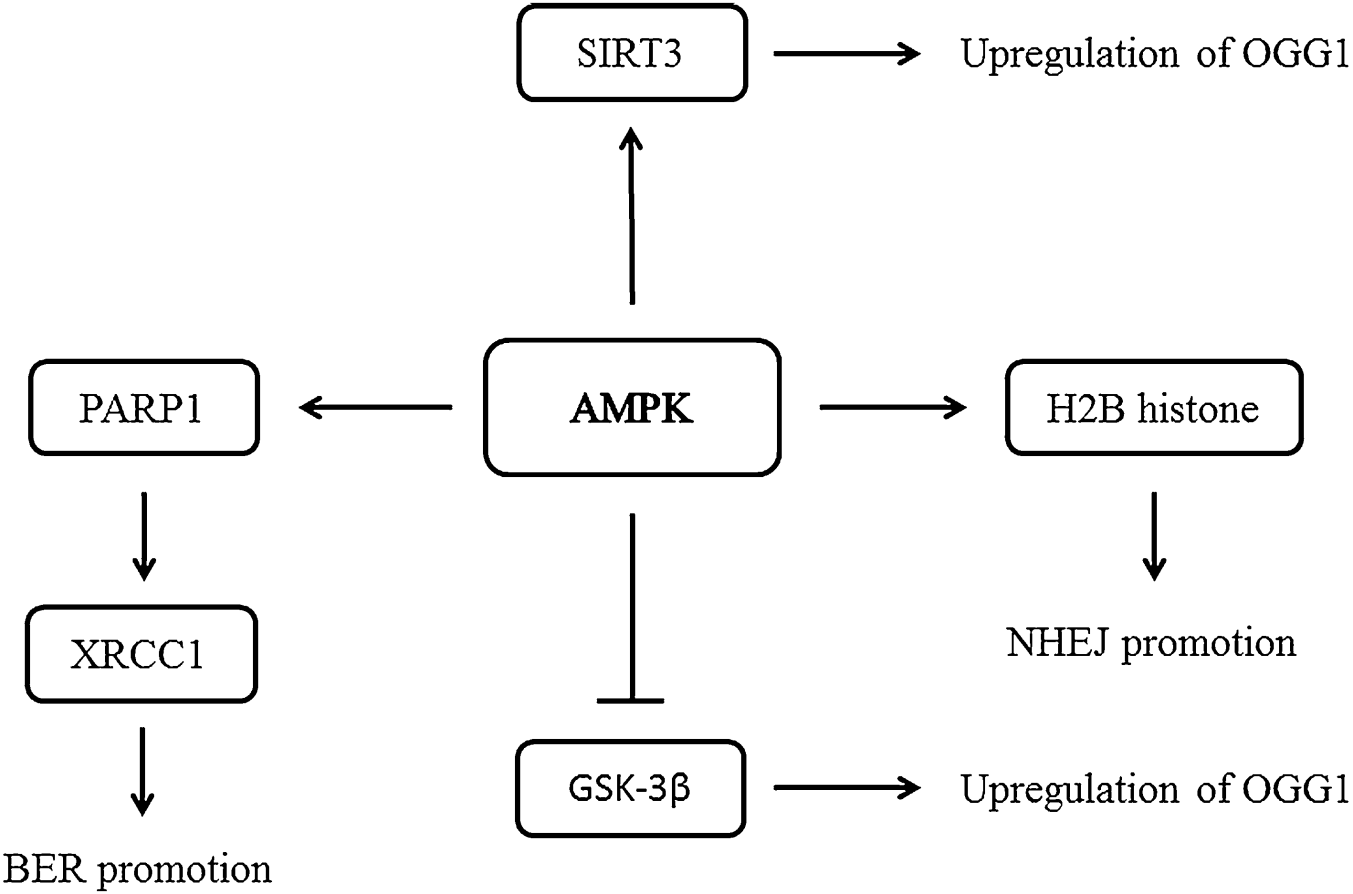
The influence of AMPK on DNA damage repair mechanisms. Examples of direct and well described actions of AMPK are shown

One of the key roles in DNA damage repair is played by the PARP superfamily. It is composed of 17 members in higher eukaryotes. The most studied protein of this superfamily is PARP-1 (poly(ADP-ribose) polymerase-1, also known as NAD^+^ ADP-ribosyltransferase 1). PARP-1 takes part in ssDNA damage repair by the BER system. PARP-1 recruits multiple XRCC1 (X-ray repair cross-complementing protein 1) proteins to a site of SSB. It is essential step before recruitment of polymerase β (Pol β) and DNA ligase III (LigIII) to the site. DNA strand breaks which induce PARP-1 activation are associated with elevated ROS generation and decreased ATP production. PARP-1 may also take part in AMPK activation through LKB1. On the other hand, AMPK is able to activate PARP-1 directly (phosphorylation) (Fig. 4). Nonetheless, AMPK activation leads to inhibition of mTOR pathway and induction of autophagy. BER system is energy-consuming—not in every case the AMPK signaling system is able to operate without being overwhelmed by massive energy balance fluctuations occurring after activation of BER pathways [95–97]. Therefore, autophagy is strongly preferred, especially after serious DNA damage.

OGG1 (8-oxoguanine glycosylase, EC 4.2.99.18) is a DNA glycosylase encoded in human by the *OGG1* gene. OGG1 is essential for the initial step of BER. It is responsible for recognition and excision of 8-oxoG from the DNA helix. Recent data show that the deficiency of OGG1 causes a 20-fold higher level of 8-oxodG in mtDNA of *OGG1* knockout mice in comparison to wild type mice. Moreover, 8-oxodG is 3 times more likely to occur in mtDNA than in nuclear DNA [9, 41, 98, 99]. There is a relationship between impaired expression of *OGG1* and carcinogenesis in eukaryotes. Research conducted on *OGG1* knockout mice have shown a significant increase in spontaneous lung adenoma/carcinoma and UV light-induced skin cancer in comparison to wild-type mice. Some studies imply similar mechanism of action in human carcinogenesis [67].

To understand the influence of AMPK signaling pathways on DNA damage repair through OGG1, a few examples should be considered. OA (osteoarthritis) is one of the diseases closely related to AMPK functionality, in particular to disturbed mitochondria functionality. OA leads to progressive degeneration of articular cartilage, which is associated with mtDNA damage in chondrocytes. They demonstrate decreased ATP generation, increased mitochondrial apoptosis and higher susceptibility to OS. Although oxidation of guanine residues to 8-oxodG plays important role in OA-related mitochondrial dysfunction, there is also evidence showing that activation of AMPK leads to elevated activity of SIRT3 (NAD-dependent deacetylase sirtuin-3). SIRT3 reduces acetylation of OGG1 and promotes its expression in OA chondrocytes, thus accelerates 8-oxodGTP removal. SIRT3 protects OGG1 from degradation since acetylated form can be easily degraded. On the other hand, SIRT3 triggers mitophagy in OA chondrocytes [98, 100]. Since in diabetes, SIRT3 expression in muscles may be significantly reduced, AMPK activation becomes even more crucial in order to modulate OGG1 activity [101, 102]. Diabetes-related decrease in SIRT3 activity may be improved by the presence of resveratrol—one of the AMPK activators [103].

Studies conducted on human dopaminergic neuroblastoma cells showed decreased expression and activity of OGG1 during long-term exposure to high glucose concentration. The effect aggravates after cell exposure to bupivacaine—a compound that enhances DNA damaging process. In non-stress conditions bupivacaine-induced DNA damage associated with decreased ATP production and increased ROS generation, causes cellular response—elevated OGG1 expression. However, in the case of long-term exposure to an increased glucose level the effect was opposite [9].

Decreased OGG1 expression is associated with Akt. Although Akt acts as agent lowering blood glucose level, there is another pathway involved in response to high glucose. An active form of Akt deactivates tuberin through its phosphorylation at Thr1462. OGG1 expression is regulated by tuberin and Akt activation is responsible for decreased expression and activity of OGG1. AMPK affecting IRS-1 and Akt-mTORC2 upregulates glucose uptake, but also creates side effect in the form of tuberin deactivation. Fortunately, in some cases tuberin-related OGG1 deactivation can be compensated by AMPK-SIRT3 pathway [27, 41, 42, 73]. Studies confirm positive influence of AMPK on OGG1 expression. In diabetic and tuberin-deficient mice, as well as in tuberin-negative mouse embryonic fibroblasts, AMPK activity and OGG1 expression are elevated by AICAR, while inhibition of AMPK results in the opposite effect [73, 104].

## Conclusions

Incidence of metabolic syndrome-related diseases is increasing worldwide. Daily dietary habits have a huge impact on the overall health condition of the human body. AMPK is the main factor regulating metabolic pathways in cells. Numerous natural plant compounds enhancing energy metabolism, due to their connection with AMPK, are now believed to support conventional therapeutic treatment of people affected by diabetes and/or abdominal obesity. Mechanisms which control energy balance in cells’ response to stress cannot fully prevent the negative effect of metabolic stress on cells’ functions and components. The same applies to DNA damage—recent studies confirm that AMPK regulates DNA repair systems and other processes determining the fate of cells such as autophagy and apoptosis. Therefore, AMPK has become the main goal of research aimed at developing combined therapeutic techniques in order to combat, among other things, the process of cancerogenesis, and which may be supported by natural nutrients, included in everyday diet. Moreover, connections between AMPK signaling pathways and cellular response to DNA damage, described in this review, may seem tenuous and need further investigation. Nevertheless, we strongly believe that the content of this review can help with research in the field of cellular response on DNA damaging factors.
